# Barriers and facilitators to implementing Decision Boxes in primary healthcare teams to facilitate shared decisionmaking: a study protocol

**DOI:** 10.1186/1472-6947-12-85

**Published:** 2012-08-06

**Authors:** Anik Giguere, Michel Labrecque, Roland Grad, Michel Cauchon, Matthew Greenway, France Légaré, Pierre Pluye, Stephane Turcotte, Lisa Dolovich, R Brian Haynes

**Affiliations:** 1 Health Information Research Unit, Department of Clinical Epidemiology and Biostatistics, McMaster University, CRL-139, 1280 Main Street West, Hamilton, ON, L8S 4 K1, Canada; 2 Research Center of the CHUQ, Saint-Francois d'Assise Hospital, 10 rue de l'Espinay, D6-730, Quebec City (QC), G1L 3 L5, Canada; 3 Department of Family Medicine, McGill University, 515-517 Pine Avenue West, Montreal (QC), H2W 1 S4, Canada; 4 Dept. of Family and Emergency Medicine, Laval University Laval, Pavillon Ferdinand-Vandry, 1050 avenue de la Médecine, Local 4617, Québec (QC), G1V 0A6, Canada; 5 Department of Family Medicine, McMaster University, 118 Lake Street, Saint-Catharines, ON, Canada; 6 Research Center of the CHUQ, Saint-Francois d'Assise Hospital, 10 rue de l'Espinay, D6-730, Quebec City (QC), G1L 3 L5, Canada; 7 Department of Family Medicine, McGill University, 515-517 Pine Avenue West, Montreal (QC), H2W 1 S4, Canada; 8 Research Center of the CHUQ, Saint-Francois d'Assise Hospital, 10 rue de l'Espinay, D6-730, Quebec City (QC), G1L 3 L5, Canada; 9 Department of Family Medicine, McMaster University, McMaster Innovation Park, 175 Longwood Road South, Hamilton, ON, L8P 0A1, Canada; 10 Department of Clinical Epidemiology and Biostatistics and Department of Medicine, DeGroote School of Medicine, McMaster University, 1280 Main Street West, CRL-125, Hamilton, ON, Canada

**Keywords:** (3–10), Evidence-based practice, Continuing professional education, Risk communication, Patient-centered care, Counselling, Clinical topic summary, Decision support, Knowledge translation, Implementation science

## Abstract

**Background:**

Decision Boxes are summaries of the most important benefits and harms of health interventions provided to clinicians before they meet the patient, to prepare them to help patients make informed and value-based decisions. Our objective is to explore the barriers and facilitators to using Decision Boxes in clinical practice, more precisely factors stemming from (1) the Decision Boxes themselves, (2) the primary healthcare team (PHT), and (3) the primary care practice environment.

**Methods/design:**

A two-phase mixed methods study will be conducted. Eight Decision Boxes relevant to primary care, and written in both English and in French, will be hosted on a website together with a tutorial to introduce the Decision Box. The Decision Boxes will be delivered as weekly emails over a span of eight weeks to clinicians of PHTs (family physicians, residents and nurses) in five primary care clinics located across two Canadian provinces. Using a web-questionnaire, clinicians will rate each Decision Box with the Information Assessment Method (cognitive impacts, relevance, usefulness, expected benefits) and with a questionnaire based on the Theory of Planned Behavior to study the determinants of clinicians’ intention to use what they learned from that Decision Box in their patient encounter (attitude, social norm, perceived behavioral control). Web-log data will be used to monitor clinicians’ access to the website. Following the 8-week intervention, we will conduct semi-structured group interviews with clinicians and individual interviews with clinic administrators to explore contextual factors influencing the use of the Decision Boxes. Data collected from questionnaires, focus groups and individual interviews will be combined to identify factors potentially influencing implementation of Decision Boxes in clinical practice by clinicians of PHTs.

**Conclusions:**

This project will allow tailoring of Decision Boxes and their delivery to overcome the specific barriers identified by clinicians of PHTs to improve the implementation of shared decision making in this setting.

## Background

Shared decision making (SDM) is an approach where healthcare professionals and patients make joint decisions based on the best evidence of the benefits and harms of all available options, and patients’ values and preferences in regard to those options [1]. Patient Decision Aids are the tools most often used to facilitate SDM. They provide patients with information on the options and research-based outcomes relevant to health status, and help to clarify values regarding the benefits and harms of each option [2]. They have been associated with favorable outcomes such as greater knowledge, lower decisional conflict, and lower proportion of people passive in decision making or undecided [2]. Despite that Patient Decision Aids help them make informed decisions, many patients are still turning to their healthcare professional for recommendations. Clinician knowledge and recommendations are recognized as essential elements of SDM [3], but clinicians often lack the tools to translate their knowledge to their patient and facilitate patients’ involvement in decision making.

To prepare healthcare professionals for SDM, we have involved both professionals and patients in the development of short clinical summaries, named ‘Decision Boxes’, that integrate the best available evidence from studies and knowledge syntheses to provide quantitative information on management options for medical questions that have no single best answer [4,5]. The Decision Box is intended to help the clinician recognize that a decision needs to be shared with the patient, to prepare the clinician to communicate evidence-based information to the patient, and to assist the clinician in seeking patient’s values and preferences regarding the decision to be made.

Despite broad recognition of the merits of SDM, there is scarce evidence that health professionals put SDM approach into practice [6-8]. The barriers to the implementation of SDM in clinical practice most often reported by health professionals are time pressure and the perception that SDM cannot be applied because of the patient’s characteristics or clinical situation [9]. We developed Decision Boxes to try to address these barriers, for example by using a brief 2-page format, by offering prompts to initiate a discussion with the patient, and by providing first-hand evidence on decisions commonly encountered in primary care [4]. However, we lack evidence on clinicians’ perceptions of the Decision Box, and on the best way to implement Decision Boxes within primary healthcare teams (PHTs).

### Objectives

Our objective is to study the barriers and facilitators influencing Primary Healthcare Teams’ uses of Decision Boxes in clinical practice. Our specific questions are: (1) What are clinicians' perceptions of the usefulness of the Decision Box? (2) Do clinicians intend to use information from Decisions Boxes in clinical practice? (3) What aspects of the primary care practice environment affect the use in practice of what clinicians learned from Decisions Boxes?

### Theoretical underpinnings

**Figure 1:**
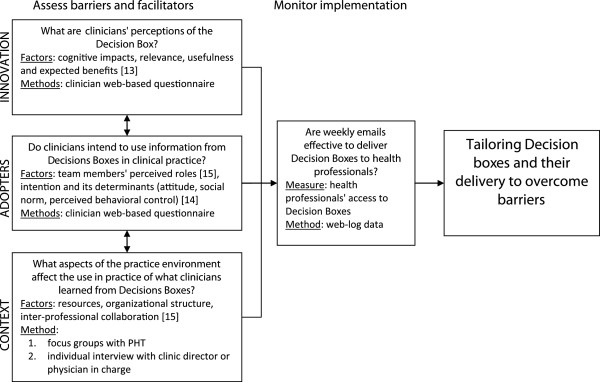
**Conceptual framework for the study of the implementation of Decision Boxes in primary healthcare teams (PHT), including factors explored and methods used.** Adapted from Graham and Logan [10].

The proposed project is based on the theory of mechanisms of planned change as described in the Ottawa Model of Research Use (OMRU) [10,11] (Figure 1).Derived from evidence and theories of change, the OMRU recognizes that practice change is not a linear process, but involves simultaneous and interactive relationships between the nature of the innovation, the potential adopters, and the context within the practice environment. Three key processes involved are: 1) assessing barriers and supports; 2) developing and monitoring interventions tailored to barriers and supports; 3) evaluating outcomes. The underlying mechanism is that tailoring intervention strategies to address barriers and strengthen facilitators related to the innovation (here Decision Box), potential adopters (here PHT) and practice environment (here primary care clinics) will result in practice change. Barriers and supports related to the innovation will be assessed with the Information Assessment Method [12], those relative to potential adopters using Ajzen's Theory of Planned Behavior (TPB) (attitude, subjective norms, perceived behavioral control) [13] and the Inter-Professional Shared Decision-Making (IP-SDM) model [14,15], and those relative to the primary care practice environment using the IP-SDM model [16]. We will use the identified barriers to tailor the Decision Box intervention before testing it in a future RCT. 

## Methods

### Study design

**Figure 2:**
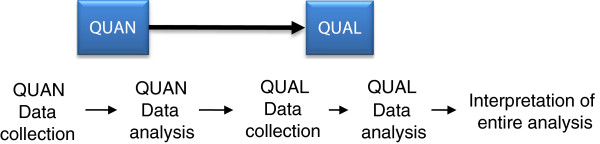
**Proposed two-phase sequential explanatory study design.** In the first phase, quantitative (QUAN) data are collected via web questionnaires and analyzed to inform second phase. In the second phase, qualitative (QUAL) data are collected via interviews and analyzed. Both types of data are then interpreted together.

We will use a two-phase mixed methods study with an explanatory sequential design [17], characterized by the collection and analysis of quantitative data in the first phase of research, followed by the collection and analysis of qualitative data in the second phase that builds on and explains the initial results of the quantitative component (Figure 2) [17]. In the first phase, a previously validated questionnaire [4] will be used to assess barriers and facilitators relative to the Decision Boxes as innovation. In the second phase, due to a lack of validated questionnaires, a qualitative study will be conducted to explain the barriers and facilitators relative to the environmental context. Quantitative and qualitative study findings will be interpreted together to adapt the Decision Boxes and the strategy to facilitate their use in clinical practice. 

### Intervention

The intervention will consist of two elements: (1) eight evidence-based Decision Boxes written in both French and English on common primary care diagnostic, therapeutic and preventive interventions, and (2) a website hosting the Decision Boxes and a brief web-based tutorial to introduce the Decision Box. Each week, one of eight Decision Boxes (Table 1) will be delivered by email to the recruited clinicians for a total of eight weeks. Each email will also contain links to (1) the web-based tutorial, and (2) a web-based questionnaire for assessing each Decision Box.

**Table 1:** Interventions covered by Decision Boxes

Screening	Box 1: 4. The serum integrated test to screen women for fetal trisomy 21
	Box 2: The BRCA1/2 gene mutation test to evaluate the risks of breast and ovarian cancer
	Box 3: The fecal occult blood test (FOBT) to screen for colorectal cancer
	Box 4: The prostate-specific antigen (PSA) test to screen men for prostate cancer
Prevention	Box 5: Acetylsalicylic acid (ASA) for primary prevention of cardiovascular disease
	Box 6: Bisphosphonates to prevent osteoporotic fractures in postmenopausal women
	Box 7: Statins for primary prevention of cardiovascular disease
Treatment	Box 8: Cholinesterase inhibitors to reduce the symptoms of Alzheimer’s disease

### Participants and recruitment strategies

 Five primary care clinics (such as FMU – Family Medicine Units - in Quebec and FHT- Family Health Team- in Ontario) will be recruited through professional networks of the members of the research team. They will include two French-speaking clinics and three English-speaking clinics. We will then invite all family physicians, nurses and residents working at those clinics to participate in the first phase of the study.

For phase two, a purposeful sample of eight clinicians per site, comprising 5 physicians, two nurses and one resident, will be randomly selected among extreme cases on the web-based questionnaires, i.e. clinicians who gave higher or lower scores to the Decision Boxes rated with the web-questionnaire. Thus, only the participants who completed at least one of the eight study questionnaires during phase one will be eligible to participate to phase 2. At each study site, we will also recruit a clinician with administrative responsibilities, such as the clinic Medical Director.

We will offer monetary compensation to the clinicians (CAN$100) who participated to the study interviews.

### Data collection and procedures

1. Phase one: quantitative study

At study entry, all participating clinicians will sign an informed consent form and complete a questionnaire assessing their demographic and professional characteristics (age, gender, number of years of clinical practice). They will also rate their interest for each of the eight Decision Box topics using a visual analogue scale ranging from "no interest" to "great interest". Then, clinicians will receive the eight Decision Boxes by email, at a rate of one per week for eight weeks. For each Decision Box received, they will be prompted to complete a web-based questionnaire (Additional file 1). The first part of this questionnaire consists of a 5-point smiley-faces rating scale to assess global satisfaction with that Decision Box. The second part consists of questions based on the Theory of Planned Behavior (TPB) [13] to study the characteristics of the clinicians that influence their intention to perform a given behavior. In this case, the behavior under study was ‘to use what I learned from the Decision Box to precisely explain the advantages and disadvantages of the options to my next patient to whom this intervention might apply’. Based on the TPB, the three determinants of a clinician’s intention to perform a behavior are: his/her attitude toward performing this behavior (perceived advantages and disadvantages of performing a behavior), his/her subjective norm (normative beliefs about the social pressure to engage or not to engage in this behavior), and his/her perceived behavioral control (beliefs, based on experience, about one’s ability to adopt this behavior). The third part of the questionnaire comprises the Information Assessment Method (IAM) that serves to evaluate cognitive impacts of the information, its relevance, usefulness and expected benefits [12].

2. Phase 2: qualitative study

At each site, we will conduct a 90-minute semi-structured interview with a mixed group comprising five family physicians, two nurses and one resident, and a 30-minute individual interview with a clinician with administrative responsibilities, such as the Medical Director of the clinic. These interviews will assess in-depth understanding of the implementation process and explore the influence of the practice environment on the clinical application of the knowledge which clinicians acquired from the Decision Boxes. Both the interview guides for individual and group interviews will be based on the conceptual model for IP-SDM [14], and cover all key topics for all focus groups, although not necessarily in the same order (interviews will follow the natural progression of the conversation). This interview format will give participants the flexibility to explore emerging issues. All interviews will be moderated by the same interviewer (AG). One observer will take notes on the process and content of the discussions. Each discussion will be audiotaped and professionally transcribed.

## Analysis plan and samples sizes

1. Phase one: quantitative study

We will first perform descriptive statistical analyses of the answers to the web-questionnaires. We will then use a mixed model to describe clinicians’ perceptions of the attributes of the decision boxes (5-point smiley-faces rating scale, IAM), taking into account the clinical topic of the Decision Box, the study site, clinician’s status (nurse, resident, physician) and socio demographic characteristics of the participants. We will also perform multiple regression analyses to test relationships between clinicians’ intention to use what they learned from the Decision Boxand the determinants of their intention (attitude, subjective norm, perceived behavioral control), their status (nurse, resident, physician), their sociodemographic characterisitics (age, gender, number of years in practice), the study site, and the clinical topic of the Decision Box.

The regression analysis between clinicians’ intention and its determinants was used to set the needed sample size of clinicians. Earlier studies estimated regression coefficients of 0.78 for physicians [18], and of 0.63 for nurses [19] between intention and the three determinants of the intention (attitude, behavioral control and subjective norm). To be conservative, we used the lowest regression coefficient obtained for nurses (R^2^ = 0.63) to calculate the sample size, using two potential confounding variables (clinical topics and clinicians’ status). The ‘clinic’ effect was considered negligible as we have previously measured an Intraclass correlation coefficient of only 0.02 for clinicians nested within clinics in a cluster randomized controlled trial [20]. Using these parameters we evaluated that a sample size of 78 clinicians gave a power of 90%. Based on a study of a continuing medical education program in shared decision-making where 70% of the physicians attended at least two of the three workshops [21], we expect that about 70% of participating clinicians will complete at least half of the eight online questionnaires (1 questionnaire/Decision Box; 8 Decision Boxes). Consequently, to achieve a sample size of at least 78 clinicians who will complete at least four questionnaires, we need to recruit 111 clinicians (n = 5 clinics; 22 clinicians/clinic).

2. Phase 2: qualitative study

Two research professionals will independently perform a hybrid deductive/inductive thematic qualitative data analysis for each group and individual interview using specialized software (NVivo 9). Any disagreements will be discussed until consensus is reached. The coding scheme will be developed following the OMRU [10,11], the Steps of the users' experience of an evidence-based shared decision-making support tool over time [4], and the IP-SDM model [14-16], by identifying what the PHT and the administrators of the primary care organizations experienced as barriers or facilitators to the application of what clinicians learned from the Decision Boxes. The deductive thematic analysis will apply attributes derived *a priori* from these theories and an inductive thematic analysis will integrate new themes, suggested by the data, into the scheme. We will compare the phenomena observed to emphasize a common tangent and will work out tree structures and matrices for the analysis. The first author (AG),will corroborate the findings by scrutinizing the analysis and ensuring that new themes, tree structures and matrices are representative of the initial data analysis and codes assigned.

We will combine data from phases one and two to identify the factors potentially influencing the implementation of the Decision Boxes in primary care. We will then tailor the implementation strategy to overcome the identified barriers and take into account the specific situation.

Approval of ethics for this project were given by the research ethics committee of the Centre de Recherche du Centre Hospitalier Universitaire de Quebec (reference number #S11-12-143), by the Research Ethic committee of the Jewish General Hospital in Montreal (reference number #12-014), and by the Research Ethics Board of McMaster University (reference number #11-550).

## Discussion

This project aims to study the factors that influence the use of a novel tool to facilitate SDM by primary healthcare teams. Factors related to the innovation itself, the potential users, and the context of use will be assessed.

Delivery of this tool in naturalistic settings will allow assessment of contextual barriers to uptake, and will help collect realistic perceptions on the value of the tool. Because eight Decision Boxes on various topics will be delivered, this project also represents a unique opportunity to explore the influence of the clinical topic on clinicians’ intention to use what they learned from the Decision Boxto explain the advantages and disadvantages of the options to their patients. The Decision Boxes will be written in both the official languages of Canada, French and English, allowing a wider dissemination in the country. As the study will include professionals from three cities located across two provinces in Canada, results will then also be generalizable to a wider population. However, the clinicians we will recruit may not be representative of all practicing physicians in the targeted settings - only those interested in Evidence-Based Medicine and/or SDM are likely to accept participating.

The Decision Box is a new tool available to clinicians who decide to use SDM to make a decision with their patient. A review of interventions to improve health professionals’ adoption of SDM in clinical practice suggest that, both patient-mediated interventions, such as Patient Decision Aids, and training of health professionals are important to the successful implementation of SDM in clinical practice [22]. As a self-directed learning approach, the Decision Box may complement group learning activities already available to teach shared decision-making [23].

Additional benefits of this project include the development of a website hosting the Decision Boxes, further facilitating access for clinicians, researchers, and patients.

This project will allow tailoring of Decision Boxes and their delivery to overcome the specific barriers for change identified in primary care clinicsin Canada. By helping implement Decision Boxes in clinical practice, this study may potentially enhance the transfer of scientific data to healthcare professionals, and also improve communication between healthcare professionals and patients, thus allowing a more judicious use of the current best available evidence in clinical decision making.

## Competing interests

The authors declare that they have no competing interests.

## Authors’ contribution

AG conceived this study with BH, RG, PP, FL, ML, and ST. AG wrote the first draft and all authors critically revised the manuscript and approved its final version.

## Pre-publication history

The pre-publication history for this paper can be accessed here:

http://www.biomedcentral.com/1472-6947/12/85/prepub

## Supplementary Material

Additional file 1 Web-based questionnaire for clinicians on their perception of the Decision Boxes (Parts 1 and 3) and on their intention to use what they learned from the Decision Box to precisely explain the advantages and disadvantages of the options to their next patient to whom this intervention might apply (Part 2). Click here for file

## Author details

^1^Health Information Research Unit, Department of Clinical Epidemiology and Biostatistics, McMaster University, CRL-139, 1280 Main Street West, Hamilton, ON L8S 4 K1, Canada. ^2^Research Center of the CHUQ, Saint-Francois d'Assise Hospital, 10 rue de l'Espinay, D6-730, Quebec City (QC) G1L 3 L5, Canada.^3^Department of Family Medicine, McGill University, 515-517 Pine Avenue West, Montreal (QC) H2W 1 S4, Canada. ^4^Dept. of Family and Emergency Medicine, Laval University Laval, Pavillon Ferdinand-Vandry, 1050 avenue de la Médecine, Local 4617, Québec (QC) G1V 0A6, Canada. ^5^Department of Family Medicine, McMaster University, 118 Lake Street, Saint-Catharines, ON, Canada. ^6^Research Center of the CHUQ, Saint-Francois d'Assise Hospital, 10 rue de l'Espinay, D6-730, Quebec City (QC) G1L 3 L5, Canada. ^7^Department of Family Medicine, McGill University, 515-517 Pine Avenue West, Montreal (QC) H2W 1 S4, Canada. ^8^Research Center of the CHUQ, Saint-Francois d'Assise Hospital, 10 rue de l'Espinay, D6-730, Quebec City (QC) G1L 3 L5, Canada.^9^Department of Family Medicine, McMaster University, McMaster Innovation Park, 175 Longwood Road South, Hamilton, ON L8P 0A1, Canada.^10^Department of Clinical Epidemiology and Biostatistics and Department of Medicine, DeGroote School of Medicine, McMaster University, 1280 Main Street West, CRL-125, Hamilton, ON, Canada.

## References

[B1] Charles C Gafni A Whelan T Decision-making in the physician-patient encounter: revisiting the shared treatment decision-making model Soc Sci Med 1999 49 651 661 10.1016/S0277-9536(99)00145-8 10452420

[B2] Stacey D Bennett CL Barry M Col NF Eden KB Holmes-Rovner M Llewellyn-Thomas H Lyddiatt A Légaré F Thomson R Decision aids for people facing health treatment or screening decisions Cochrane Database Syst Rev 2011 5 10 CD001431 21975733 10.1002/14651858.CD001431.pub3

[B3] Makoul G Clayman ML An integrative model of shared decision making in medical encounters Patient Educ Couns 2006 60 301 312 10.1016/j.pec.2005.06.010 16051459

[B4] Giguere A Légaré F Grad R Pluye P Haynes R Cauchon M Rousseau F Argote J Labrecque M Decision Boxes for clinicians to support evidence-based practice and shared decision making: the user experience Implem Sci 2012 7 72 10.1186/1748-5908-7-72 PMC3533695 22862935

[B5] Giguere A Legare F Grad R Pluye P Rousseau F Haynes RB Cauchon M Labrecque M Developing and user-testing Decision boxes to facilitate shared decision making in primary care - a study protocol BMC Med Inform Decis Mak 2011 11 17 10.1186/1472-6947-11-17 21385470 PMC3060840

[B6] Edwards A Elwyn G Wood F Atwell C Prior L Houston H Shared decision making and risk communication in practice: a qualitative study of GPs' experiences Br J Gen Pract 2005 55 6 13 15667759 PMC1266236

[B7] Edwards M Davies M Edwards A What are the external influences on information exchange and shared decision-making in healthcare consultations: A meta-synthesis of the literature Patient Educ Couns 2009 75 37 52 10.1016/j.pec.2008.09.025 19036550

[B8] Holmes-Rovner M Valade D Orlowski C Draus C Nabozny-Valerio B Keiser S Implementing shared decision-making in routine practice: barriers and opportunities Health Expect 2000 3 182 191 10.1046/j.1369-6513.2000.00093.x 11281928 PMC5080967

[B9] Legare F Ratte S Gravel K Graham ID Barriers and facilitators to implementing shared decision-making in clinical practice: update of a systematic review of health professionals' perceptions Patient Educ Couns 2008 73 526 535 10.1016/j.pec.2008.07.018 18752915

[B10] Graham ID Logan J Innovations in knowledge transfer and continuity of care The Canadian journal of nursing research= Revue canadienne de recherche en sciences infirmières 2004 36 89 15369167

[B11] Graham K Logan J Using the Ottawa Model of Research Use to Implement a Skin Care Program J Nurs Care Qual 2004 19 18 26 10.1097/00001786-200401000-00006 14717144

[B12] Pluye P Grad RM Johnson-Lafleur J Bambrick T Burnand B Mercer J Marlow B Campbell C Evaluation of email alerts in practice: Part 2 - validation of the information assessment method J Eval Clin Pract 2010 16 1236 1243 10.1111/j.1365-2753.2009.01313.x 20722882

[B13] Ajzen I The theory of planned behavior Organ Behav Hum 1991 50 179 211 10.1016/0749-5978(91)90020-T

[B14] Legare F Stacey D Gagnon S Dunn S Pluye P Frosch D Kryworuchko J Elwyn G Gagnon MP Graham ID Validating a conceptual model for an inter-professional approach to shared decision making: a mixed methods study J Eval Clin Pract 2011 17 554 564 10.1111/j.1365-2753.2010.01515.x 20695950 PMC3170704

[B15] Legare F Stacey D Pouliot S Gauvin FP Desroches S Kryworuchko J Dunn S Elwyn G Frosch D Gagnon MP Interprofessionalism and shared decision-making in primary care: a stepwise approach towards a new model J Interprof Care 2011 25 18 25 10.3109/13561820.2010.490502 20795835 PMC3018136

[B16] Legare F Stacey D Briere N Desroches S Dumont S Fraser K Murray MA Sales A Aube D A conceptual framework for interprofessional shared decision making in home care: protocol for a feasibility study BMC Health Serv Res 2011 11 23 10.1186/1472-6963-11-23 21281487 PMC3045286

[B17] Creswell JW Research design: Qualitative, quantitative, and mixed methods approaches 2009 Sage Publications, Inc

[B18] Legare F Graham ID O'Connor AC Aubin M Baillargeon L Leduc Y Maziade J Prediction of health professionals' intention to screen for decisional conflict in clinical practice Health Expect 2007 10 364 379 10.1111/j.1369-7625.2007.00465.x 17986073 PMC5060414

[B19] Jurgens D Nurses' intentions to administer morphine for postoperative pain: An application of Ajzen's theory of planned behaviour PhD thesis 1996 The University of Saskatchewan

[B20] Legare F Labrecque M LeBlanc A Njoya M Laurier C Cote L Godin G Thivierge RL O'Connor A St-Jacques S Training family physicians in shared decision making for the use of antibiotics for acute respiratory infections: a pilot clustered randomized controlled trial Health Expect 2011 1 14 Suppl 96 110 20629764 10.1111/j.1369-7625.2010.00616.x PMC3073122

[B21] Leblanc A Legare F Labrecque M Godin G Thivierge R Laurier C Cote L O'Connor AM Rousseau M Feasibility of a randomised trial of a continuing medical education program in shared decision-making on the use of antibiotics for acute respiratory infections in primary care: the DECISION+ pilot trial Implement Sci 2011 6 5 10.1186/1748-5908-6-5 21241514 PMC3033351

[B22] Legare F Ratte S Stacey D Kryworuchko J Gravel K Graham ID Turcotte S Interventions for improving the adoption of shared decision making by healthcare professionals Cochrane Database Syst Rev 2010 5 CD006732 20464744 10.1002/14651858.CD006732.pub2

[B23] Légaré F Inventory of Shared Decision Making Programs for Healthcare Professionals http://decision.chaire.fmed.ulaval.ca/index.php?id=180&L=2

