# Supplementary material for: Barriers and facilitators to implementing Decision Boxes in primary healthcare teams to facilitate shared decisionmaking: a study protocol
Source: BMC Med Inform Decis Mak. 2012 Aug 6;12:85. doi: 10.1186/1472-6947-12-85 (PMC3472191; doi:10.1186/1472-6947-12-85)
Supplement: Additional file 1 — Web-based questionnaire for clinicians on their perception of the Decision Boxes (Parts 1 and 3) and on their intention to use what they learned from the Decision Box to precisely explain the advantages and disadvantages of the options to their next patient to whom this intervention might apply (Part 2). [file 1472-6947-12-85-S1.pdf]

## PART I: General appreciation

1. How did you like this Decision Box?

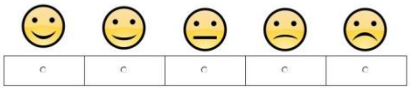

## PART II: Questionnaire based on the Theory of planned behaviour

**Now, thinking of your next patient to who this intervention might apply**, please read the following comments and check the answer of your choice.

2. I intend to use what I learned from this document to precisely explain to this patient the advantages and disadvantages of the options.

| ○ -3 | ○ -2 | ○ -1 | ○ 0 | ○ +1 | ○ +2 | ○ +3 |
|---|---|---|---|---|---|---|
| **Strongly disagree** | | | | | | **Strongly agree** |

3. Most of my patients would approve of my using what I learned from this document to precisely explain to this patient the advantages and disadvantages of the options.

| ○ -3 | ○ -2 | ○ -1 | ○ 0 | ○ +1 | ○ +2 | ○ +3 |
|---|---|---|---|---|---|---|
| **Would strongly disapprove** | | | | | | **Would strongly approve** |

It seems to me that using what I learned from this document to precisely explain the advantages and disadvantages of the options to my next patient who considers this intervention is...

4.

| ○ -3 | ○ -2 | ○ -1 | ○ 0 | ○ +1 | ○ +2 | ○ +3 |
|---|---|---|---|---|---|---|
| **Very useless** | | | | | | **Very useful** |

5.

| ○ -3 | ○ -2 | ○ -1 | ○ 0 | ○ +1 | ○ +2 | ○ +3 |
|---|---|---|---|---|---|---|
| **Very difficult** | | | | | | **Very easy** |

6.

| ○ -3 | ○ -2 | ○ -1 | ○ 0 | ○ +1 | ○ +2 | ○ +3 |
|---|---|---|---|---|---|---|
| **Very irresponsible** | | | | | | **Very responsible** |

7.

| ○ -3 | ○ -2 | ○ -1 | ○ 0 | ○ +1 | ○ +2 | ○ +3 |
|---|---|---|---|---|---|---|
| **Very unlikely** | | | | | | **Very likely** |

8.

| ○ -3 | ○ -2 | ○ -1 | ○ 0 | ○ +1 | ○ +2 | ○ +3 |
|---|---|---|---|---|---|---|
| **Very harmful** | | | | | | **Very beneficial** |

9. My close colleagues would approve of my using what I learned from this document to precisely explain to this patient the advantages and disadvantages of the options.

| | | | | | | |
|---|---|---|---|---|---|---|
| ○ **-3** | ○ **-2** | ○ **-1** | ○ **0** | ○ **+1** | ○ **+2** | ○ **+3** |
| **Strongly disagree** | | | | | | **Strongly agree** |

10. I feel able to use what I learned from this document to precisely explain to this patient the advantages and disadvantages of the options.

| | | | | | | |
|---|---|---|---|---|---|---|
| ○ **-3** | ○ **-2** | ○ **-1** | ○ **0** | ○ **+1** | ○ **+2** | ○ **+3** |
| **Strongly disagree** | | | | | | **Strongly agree** |

11. I will use what I learned from this document to precisely explain to this patient the advantages and disadvantages of the options.

| | | | | | | |
|---|---|---|---|---|---|---|
| ○ **-3** | ○ **-2** | ○ **-1** | ○ **0** | ○ **+1** | ○ **+2** | ○ **+3** |
| **Very unlikely** | | | | | | **Very likely** |

12. I see many reasons to use what I learned from this document to precisely explain to my next patient the advantages and disadvantages of the options.

| | | | | | | |
|---|---|---|---|---|---|---|
| ○ **-3** | ○ **-2** | ○ **-1** | ○ **0** | ○ **+1** | ○ **+2** | ○ **+3** |
| **Strongly disagree** | | | | | | **Strongly agree** |

13. Most people in my professional surroundings would urge me to use what I learned from this document to precisely explain to this patient the advantages and disadvantages of the options.

| | | | | | | |
|---|---|---|---|---|---|---|
| ○ **-3** | ○ **-2** | ○ **-1** | ○ **0** | ○ **+1** | ○ **+2** | ○ **+3** |
| **Strongly disagree** | | | | | | **Strongly agree** |

# PART III : Information Assessment Method (IAM)

## SECTION #1 – COGNITIVE IMPACTS of the information

**14. What is the impact of this information on you or your practice?** ***Please check all that apply***

Note: You can check more than one type of impact[1]

My practice is (will be) changed and improved ○

*If Yes, what aspect is (will be) changed or improved?*

- *Diagnostic approach?* ○
- *Therapeutic approach?* ○
- *Disease prevention or health education?* ○
- *Couselling approach?* ○

I learned something new ○

I am motivated to learn more ○

This information confirmed I did (am doing) the right thing ○

I am reassured ○

I am reminded of something I already knew ○

I am dissatisfied ○

There is a problem with the presentation of this information ○

*If Yes, what problem do you see?*

- *Too much information?* ○
- *Not enough information?* ○
- *Information poorly written?* ○
- *Too technical?* ○
- *Other?* ○
  *If yes, please describe this problem.*

| *Open text field* |
|---|

I disagree with the content of this information ○

This information is potentially harmful. ○

*If yes, please describe how this information may be harmful*

| *Open text field* |
|---|

[1] Note to programmer: MUST check at least one

## Section #2 - RELEVANCE of the information

**15. Is this information relevant for at least one of your patients?**[2]

- ○ Totally relevant
- ○ Partially relevant
- ◉ Not relevant

## Section #3 - USE of the information

| **16. Will you use this information for a specific patient?**[3] | **Yes** | **No** | **Possibly** |
|---|---|---|---|
| | ○ | ⊙ | ○ |

**If YES:** ***Please check all that apply***[4]
Note: You can check more than one type of use.

- As a result of this information I will manage this patient differently ○
- I had several options for this patient, and I will use this information to justify a choice ○
- I did not know what to do, and I will use this information to manage this patient ○
- I thought I knew what to do, and I used this information to be more certain about the management of this patient ○
- I used this information to better understand a particular issue related to this patient ○
- I will use this information in a discussion with this patient, or with other health professionals about this patient ○
- I will use this information to persuade this patient, or to persuade other health professionals to make a change for this patient ○

[2] Note to programmer: Answering "No" will disable Question #16
[3] Note to programmer: Answering "No" or “Possibly” will disable items of ‘use’ and question #17
[4] Note to programmer: **MUST check at least one**

## Section #4 – EXPECTED BENEFITS of the information

| **17. For this patient, do you expect any health benefits as a result of applying this information?**[5] | **Yes** | **No** |
|---|---|---|
| | ○ | ⦿ |

If YES: Check all that apply. You may check more than type of health benefit [6]

| | |
|---|---|
| This information will allow the patient to make a decision that is in better agreement with his/her personnal circumstances, values and preferences. | ○ |
| This information will help reduce the patient's uncertainty about the best decision to make. | ○ |
| This information will help to avoid unnecessary or inappropriate treatment, diagnostic procedures, preventative interventions or a referral, for this patient | ○ |

**Comment on this information or this questionnaire.**

| Open text field |
|---|

***Thank you for having completed this questionnaire***

[5] Answering "No" will disable items of 'health benefit'
[6] Note to programmer**: MUST check at least one**
